# Mitral valve abnormalities associated with single-ventricle palliation, cardiac death or transplant in fetuses with postnatally confirmed coarctation of the aorta

**DOI:** 10.21203/rs.3.rs-3272954/v1

**Published:** 2023-08-23

**Authors:** Alex J. Foy, Jonathan H. Soslow, Ann L. Kavanaugh-McHugh, Stacy A. S. Killen

**Affiliations:** Children’s Hospital and Medical Center; Vanderbilt University Medical Center; Vanderbilt University Medical Center; Vanderbilt University Medical Center

**Keywords:** Coarctation of the aorta, mitral valve, congenital heart disease, fetus, single ventricle

## Abstract

**Introduction:**

Predicting if a fetus with borderline left heart structures and coarctation of the aorta (CoA) will require single ventricle palliation (SVP) is challenging, partly due to the limitations of fetal echocardiography in defining valvar abnormalities. Fetal echocardiographic findings predictive of SVP, particularly in relation to the mitral valve (MV), are not well defined.

**Methods:**

We performed a retrospective review of fetuses with postnatally confirmed CoA from 2010 to 2020. Fetuses with complex congenital heart disease or unequivocal hypoplastic left heart syndrome were excluded. Data were compared between those who underwent biventricular repair (BVR) vs. SVP cardiac death or orthotopic heart transplant (OHT) to determine differences in fetal echocardiograms.

**Results:**

Of 67 fetuses with 131 total echocardiograms, 62 (93%) underwent BVR and 5 (7%) experienced SVP, cardiac death or OHT. Fetuses with confirmed CoA who experienced SVP cardiac death, or OHT, had fetal MV z-scores that were 2.06 lower, on average, than those who underwent BVR (z-score = −3.98 vs. −1.92, 95% CI: −2.96, −1.16). The incidences of MV anomalies and left to right flow across the foramen ovale were higher in the SVP cardiac death and OHT group.

**Conclusion:**

SVP, cardiac death or OHT in fetuses with confirmed CoA were associated with fetal MV hypoplasia, MV anomalies and left to right flow across the foramen ovale. These findings may help guide prenatal counseling about the likelihood of SVP, cardiac death or OHT in fetuses with CoA and borderline left heart structures.

## Introduction

Coarctation of the aorta (CoA) is one of the more common forms of congenital heart disease, accounting for about 8% of cardiac defects [1]. Aortic arch hypoplasia and CoA lie on a spectrum of left-sided underdevelopment, including aortic stenosis and mitral valve (MV) disease, and may be associated with an underdeveloped or hypoplastic left ventricle (LV) [2]. Hypoplastic left heart syndrome is diagnosed when the LV and associated left-sided structures are unequivocally too small to support a biventricular circulation. However, the LV and associated structures may also be borderline or mildly underdeveloped. A borderline LV has not been objectively defined but describes a ventricle that may or may not be adequate to provide systemic cardiac output [3]. Several studies have focused on the borderline fetal LV and echocardiographic measures associated with the need for neonatal intervention or single ventricle palliation (SVP) [3–7]. Unfortunately, fetal echocardiographic findings that predict SVP remain poorly defined, particularly in relation to the MV. While larger MV z-scores are associated with no postnatal intervention, z-scores ranging from less than a relatively normal value of −1.9 to a markedly hypoplastic value of −4.5 have been associated with SVP [3–7].

Prenatal counseling for fetuses with a suspected CoA and borderline LV is challenging. The false positive detection of fetal anomalies is not benign and has been shown to negatively affect the developing maternal-infant bond [8]. In the setting of nonspecific echocardiographic parameters, it can be challenging to choose when and how much to counsel about the potential for SVP. However, the difference between outcomes is stark. A neonate with CoA and an adequate LV may require only one lifetime surgical intervention while those with an inadequate LV will require a multi-stage SVP associated with high lifelong mortality, morbidity and potential need for orthotopic heart transplant (OHT). The possibility of SVP in fetuses with CoA and borderline left heart structures warrants dedicated investigation to adequately counsel parents in the prenatal period.

The objective of this study was to determine if prenatally identified MV abnormalities could predict postnatal surgical outcomes (isolated CoA repair versus SVP) in fetuses with postnatally confirmed CoA. We hypothesized that fetuses with CoA who underwent SVP postnatally had smaller and more abnormal mitral valves compared to those who underwent biventricular repair (BVR).

## Material and methods

This retrospective, single-center, cohort study was approved by the Vanderbilt Institutional Review Board prior to initiation. Vanderbilt Pediatric Heart Institute’s fetal quality assurance database, established in 2010, was queried for all fetal studies between 2010 and 2020 with aortic arch hypoplasia, CoA, or right greater than left ventricular size discrepancy. The transthoracic echocardiographic database was queried simultaneously for infants less than 60 days of age with diagnosis of CoA and compared to the fetal database for consideration (e.g., missed fetal diagnosis of CoA). Exclusionary criteria included heterotaxy syndrome, hypoplastic left heart syndrome or severe LV hypoplasia, atrioventricular septal defects, conotruncal anomalies, anomalous pulmonary venous return, and moderate or larger ventricular septal defects. Patients were also excluded for normal or absent postnatal echocardiograms. Patients with genetic and extracardiac anomalies were included in the study.

All fetal echocardiograms were performed at our institution using Acuson Sequoia, Acuson S3000 (Siemens Medical Solutions, Malvern, Pennsylvania, USA) or Phillips iE33 (Phillips Healthcare, Andover, Maryland, USA). All patients underwent a comprehensive echocardiographic examination including measurements of fetal biometry, atrioventricular valves from the hinge points in diastole, semilunar valves at the hinge points in systole, right and left ventricular end-diastolic length and width, outflow tracts and arches. All valves and outflow tracts were evaluated with pulsed-wave and color Doppler assessment, and color Doppler flow was assessed across the aortic arch, ductal arch, and foramen ovale in the sagittal view. Z-scores were calculated based on gestational age (GA) by last menstrual period using an unpublished normal measurement database from Boston Children’s Hospital (parameterz.com) [9]. Measurements obtained by the cardiologist at the initial and subsequent evaluations were used. MV measurements at the initial fetal consultation were reevaluated by a blinded fetal cardiologist (SK) as a measure of interobserver variability.

Fetal parameters collected included the absolute measurement and z-score for the atrioventricular and semilunar valves, right and left ventricular length and width, ascending aorta, and aortic isthmus (sagittal and 3-vessel). Anomalies of the MV, presence of a left superior vena cava and direction of flow across the foramen ovale were recorded. Maternal records were reviewed for demographics including GA at the initial fetal echocardiogram, obstetric comorbidities, prenatal genetic testing, presence of extracardiac anomalies, indication for referral, and prenatal counseling about the anticipated cardiac surgical course. Postnatal medical records were reviewed for cardiac diagnosis, GA at birth, birthweight, genetic testing, presence of extracardiac anomalies, recommended surgical approach and outcomes, and last available clinical status. Study data were collected and managed using REDCap (Research Electronic Data Capture) tools hosted at Vanderbilt University.

Neonatal outcomes were classified into two groups—BVR (aortic arch repair with or without additional interventions) or a composite group of SVP, cardiac death or OHT, as determined by chart review of patient notes and operative reports. Cardiac death was defined as mortality due to cardiac intervention that was not primarily explained by an alternative cause. With a variable number of studies for each fetus, MV measurements were summarized for each fetus to a single measurement targeted to estimate each fetus’ response at 29 weeks, reflecting the midpoint GA in the data set (range 20–38 weeks) [10]. Data are presented as frequency (percentage) for categorical variables, and both mean (standard deviation) and median [interquartile range] for continuous variables. Interobserver variability for initial fetal MV measurements was evaluated using Pearson’s product-moment correlation with a 95% confidence interval. Univariate comparisons were not performed due to low census in the SVP cardiac death and OHT group. A secondary univariate comparison of echocardiographic parameters between coarctation approaches (aortic arch augmentation via midline sternotomy or coarctectomy with end-to-end anastomosis via lateral thoracotomy) in the BVR group was performed using Wilcoxon rank sum test for continuous variables and Fisher’s exact test for categorical variables. A linear regression model (ordinary least squares regression) was used to estimate the mean difference between, and predicted response in fetal MV z-scores for the two surgical outcome groups. A cluster sandwich covariance estimator was used to estimate standard errors. All analyses were performed with R Version 4.2.2.

## Results

The study included 67 individual fetuses with 131 collective fetal echocardiograms. Maternal and fetal demographics are described in Table 1. Median maternal age and GA at the initial study was 28 years [IQR 23, 32] and 30.0 weeks [IQR 25.5, 33.0], respectively. Patients were referred for consultation primarily due to an abnormal obstetric cardiac ultrasound (87%). Fetuses with suspected CoA not included in the study included an in-utero demise, three stillbirths (one with trisomy 18), an elective termination, and eight neonates who passed from noncardiac causes prior to obtaining postnatal echocardiograms. CoA was not suspected by fetal echocardiogram in six patients who were diagnosed postnatally. There was a strong correlation between the fetal MV measurements at initial consultation compared with those performed by a single blinded fetal cardiologist (Pearson correlation coefficient 0.859 [95% CI: 0.780, 0.912], p<0.001).

Neonatal surgery was recommended for all patients and the median age at surgery was 7 days [IQR 5, 12]. BVR was recommended in 62 patients (93%). There were six deaths (9%) in the BVR group prior to surgery that were attributed to non-cardiac causes including congenital diaphragmatic hernia (4), trisomy 13 (1), and holoprosencephaly (1). The remaining 56 patients underwent neonatal BVR with repair of CoA; 33 (53%) underwent aortic arch augmentation via midline sternotomy, and 23 (37%) underwent coarctectomy with end-to-end anastomosis via lateral thoracotomy. There were five patients (7%) in the SVP cardiac death or OHT including two patients who underwent SVP (stage 1 Norwood palliation), two who underwent balloon aortic valvuloplasty for critical aortic stenosis (one of whom passed on ECMO following the procedure and the other who subsequently underwent OHT for poor cardiac output), and one who underwent a BVR and died at one year of age following MV revision for residual stenosis. There were no surgical conversions between single and biventricular physiologies. There were three postoperative and two late BVR deaths attributed to non-cardiac causes. The three postoperative deaths were attributed to respiratory failure in the setting of pulmonary vein stenosis, pulmonary hypertension and trisomy 21 (1), complications from tracheoesophageal fistula and a neural tube defect (1), and renal failure due to renal dysplasia (1). The two late deaths were due to multiple anomalies in the setting of Trisomy 19/Monosomy 18 (1) and presumed septic shock remote from repair (1; more than 18 months post-repair). Pathologic genetic conditions were identified in 13 patients (only in the BVR group) including Turner syndrome (6), trisomy 21 (3), trisomy 13 (1), or other (3), and were associated with one preoperative (trisomy 13) and three postoperative mortalities.

**Figure 1:**
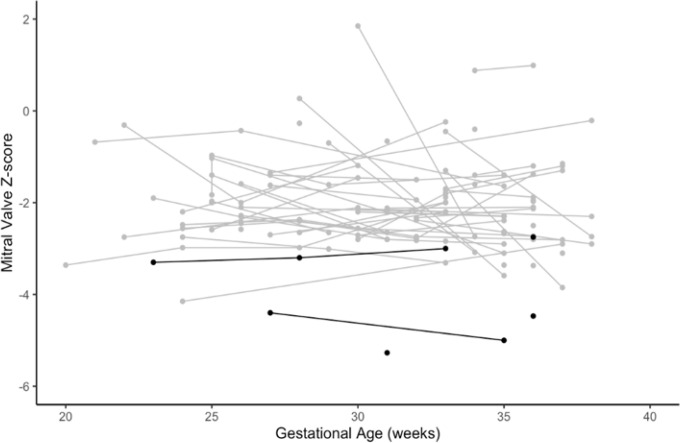
Mitral valve z-scores for each fetus by gestational age Fetuses who underwent postnatal BVR are shown in **gray**; fetuses who underwent SVP, cardiac death or OHT are shown in **black**

**Figure 2:**
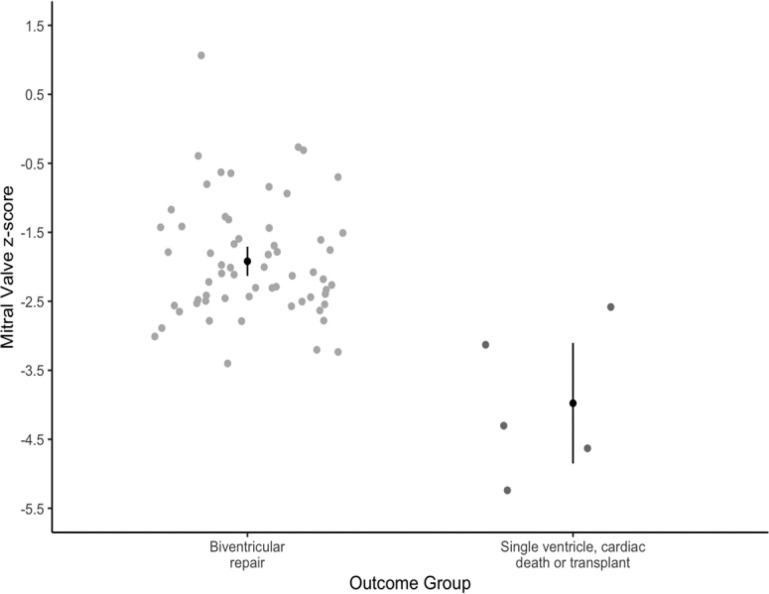
Comparison of predicted mitral valve z-scores using linear regression model. Linear regression model (ordinary least squares regression) to estimate mean response and standard errors in fetal mitral valve z-scores with a target time frame of 29 weeks. BVR (biventricular repair): z-score = −1.92 (95% CI: −2.13, −1.71). SVP (single ventricle palliation), cardiac death or OHT (orthotopic heart transplant): z-score −3.98 (95% CI: −4.85, −3.10). Compared to BVR, those in the SVP, cardiac death or OHT had a mitral valve z-score −2.06 lower, on average (95% CI: −2.96, −1.16).

Echocardiographic parameters for each outcome group are outlined in Table 2. Statistical p-values were not calculated due to the low census in the SVP, cardiac death or OHT group. The MV z-score trend for each patient was plotted against GA in Figure 1, with those in the SVP, cardiac death or OHT group highlighted in black. The predicted MV z-score for each group at 29 weeks, based on a linear regression model using an interpolated estimate, was −1.92 (95% CI: −2.13, −1.71) for the BVR group vs. −3.98 (95% CI: −4.85, −3.10) for the SVP, cardiac death or OHT group (see Figure 2). Compared to BVR, those in the SVP, cardiac death or OHT group had a MV z-score that was 2.06 lower, on average (95% CI: −2.96, −1.16). The lowest z-score associated with postnatal BVR was −4.15. No other z-scores in the BVR group were less than −4.0. In a subgroup analysis, CoA repair via midline sternotomy vs. lateral thoracotomy was not associated with differences in fetal MV z-score or other echocardiographic parameters.

Prenatally there were 43 patients (69%) in the BVR group with abnormal mitral valves, including 41 with hypoplastic annulus measurements (z-score less than −2.0). Abnormal mitral morphology was noted in four fetuses including three with suspected parachute valves and two with dysplastic or restricted leaflets. Isolated MV dysfunction was identified in three patients, including two with insufficiency and one with restricted leaflets. All five fetuses in the SVP cardiac death or OHT group had hypoplastic mitral valves prenatally, including four with morphologic or functional differences (Table 3).

Postnatally there were 31 patients (50%) in the BVR group with abnormal mitral valves, including 17 (27%) with a hypoplastic annulus on initial echocardiogram (z-score less than −2.0). Abnormal mitral morphology was noted in 11 patients (18%), including four with short/thickened chordae, three with parachute valves, two with asymmetric papillary muscles, one with a supramitral ring, and one with a double-orifice MV. Isolated MV dysfunction was identified in 13 patients, including five with insufficiency, four with stenosis, and four with both stenosis and insufficiency. Four of the five patients (80%) in the SVP, cardiac death or OHT group had significant structural or functional MV abnormalities including a supramitral ring, parachute valve, arcade, and abnormal chordae and papillary muscles (Table 3). The sensitivity of fetal echocardiography in detecting MV anomalies prenatally was 87% with a false-positive and false-negative rate of 50% and 13%, respectively, when compared with postnatal echocardiograms.

Prenatal counseling was fully concordant with postnatal outcomes in 45 patients (67%). In the BVR group, 18 (29%) patients were counseled about the possibility of SVP. All patients in the SVP group were counseled about the possibility of SVP. Prenatal counseling about postnatal ventricular outcomes changed course in 11 patients (16%; between SVP, BVP, or the possibility of either) based on subsequent fetal echocardiograms with either increasing (6) or decreasing (5) confidence with respect to postnatal BVR.

## Discussion

Prenatal diagnosis of CoA remains challenging, with several studies focusing on improvements in detection [11 – 15]. When CoA is suspected in the fetus, left-sided heart features are often borderline. More significant left heart hypoplasia may lead to postnatal SVP with a 5-year survival rate of 50–69%, compared to an isolated neonatal CoA repair survival as high as 98% [16–17]. However, SVP for the borderline left heart is uncommon, approaching 7% in both ours and a comparable study [7]. These dramatic differences in survival coupled with infrequent SVP outcomes pose a counseling dilemma for the fetal cardiologist in balancing the importance of parental education against undue stress and anxiety about postnatal expectations.

Some studies seeking to identify fetal echocardiographic features predictive of postnatal surgical outcomes have included a heterogeneous group of congenital heart lesions including unbalanced atrioventricular septal defects, conotruncal defects, aortic stenosis and hypoplastic left heart syndrome [4, 6]. Others have focused on cohorts with isolated LV hypoplasia [3, 5, 7]. Our study included all fetuses with postnatally confirmed CoA, absent other major congenital heart disease, who required surgical intervention postnatally. Despite often being considered a separate entity from left heart hypoplasia, fetuses with critical aortic stenosis were included due to similarities in prenatal and postnatal course [18–21].

Fetal MV z-scores associated with SVP in other studies have ranged from less than a low normal of −1.9 to more significantly hypoplastic scores of less than − 3.6 or −4.5 [3, 5–7]. Some of these z-score “cut-offs” were combined with left to right flow across the foramen ovale, LV length z-score, or other parameters to increase sensitivity or statistical significance [5, 7]. None of these studies specifically evaluated the association of morphologic or functional differences in the MV on postnatal outcomes. Wide ranging z-scores are difficult to apply clinically, leading some centers to counsel most or all patients with even mildly hypoplastic left heart structures about the possibility of SVP [22]. In our cohort, nearly a third of parents (29%) in the BVR group were counseled about the possibility of SVP. Additionally, there was a shift in prenatal counseling in a significant number of cases (16%), further reflecting the uncertainty in diagnosis.

In this study, those in the SVP, cardiac death or OHT outcome group had a significantly different predicted MV z-score that was 2.06 lower, on average, than the BVR group (z-score = −3.98 vs. −1.92, 95% CI: −2.96, −1.16). Morphologic and functional differences of the MV were also more common in those who experienced SVP, cardiac death or OHT (80% vs. 11 %), as well as being generally prevalent across the entire cohort (16%). Fetal echocardiography was relatively sensitive in detecting anomalies of the MV, but with a high false-positive rate. There was also less MV hypoplasia noted postnatally, likely due to differences between fetal and postnatal physiology (e.g., increased LV preload postnatally).

Left to right or bidirectional flow across the foramen ovale was more prevalent in the SVP, cardiac death or OHT group (100% vs. 27%). There were no fetuses with consistent right to left flow across the foramen ovale that experienced SVP, cardiac death or OHT, similar to previous studies reflecting the low likelihood of these patients to experience SVP [5, 7]. Although our study excluded nonsurgical patients, most fetuses who underwent a BVR had normal right to left flow across the foramen ovale throughout gestation (69%), suggesting that an abnormal flow pattern across the foramen ovale may not be as sensitive in predicting isolated CoA as previously observed [13].

Our cohort was restricted to fetuses who required postnatal cardiac surgery and was not designed to discriminate parameters for those who did not require intervention (e.g., predicting CoA). There was a limited number of SVP, cardiac death and OHT outcomes which precluded traditional univariate and multivariate analyses. MV measurements were specifically evaluated using linear regression given the primary aims of this study, but this technique was not broadly applied to other measurements due to statistical limitations. Further studies with larger sample sizes are indicated to evaluate other echocardiographic parameters.

The usual limitations of a retrospective, single center study apply. Fetal measurements were obtained over the course of a decade, concurrent with advancements in ultrasound technology and changes in fetal imaging protocols. Echocardiographic measurements were used as reported in the original studies with interobserver variability in MV measurements as reported for the initial studies only. Observations in the echocardiogram studies were subject to the interpretation of the original observers, including the degree of left ventricular hypoplasia. While z-scores allow for the normalization of measurements to GA, their use has known limitations [7, 23, 24]. Surgical recommendations were determined after birth, with candidacy for BVR subject to the interpretation of cardiologist and surgeon. The findings identified in this study have not been evaluated prospectively but should be validated in future studies.

## Conclusion

SVP, cardiac death or OHT in fetuses with confirmed CoA was associated with fetal MV hypoplasia, MV anomalies and left to right flow across the foramen ovale. Prenatal MV z-scores for fetuses in this group were, on average, 2.06 lower than for fetuses who underwent postnatal BVR. Z-scores for fetuses undergoing BVR were as low as −4.15. These findings may help guide prenatal counseling about the likelihood of SVP, cardiac death or OHT in fetuses with CoA and borderline left heart structures.

## Figures and Tables

**Table 1.** Maternal and fetal demographics

	n	Total n = 67	n	Biventricular repair n = 62	n	SVP, cardiac death or OHT n = 5

Maternal age	67	28 (±6)	62	29 (±6)	5	25 (±4)
		28 [23, 32]		28 [23, 33]		26 [21, 27]
Maternal race	67		62		5	
AA/Black		5 (7%)		4 (6%)		1 (20%)
Caucasian/White		53 (79%)		50 (81%)		3 (60%)
Hispanic/Latino		6 (9%)		5 (8%)		1 (20%)
Other		3 (4%)		4 (6%)		0 (0%)
Indication for referral	67		62		5	
Abnormal cardiac US		58 (87%)		53 (85%)		5 (100%)
Congenital anomaly		6 (9%)		6 (10%)		0 (0%)
Family history CHD		2 (3%)		2 (3%)		0 (0%)
Fetal arrhythmia		1 (1%)		1 (2%)		0 (0%)
GA at referral	67	29.5 (±4.6)	62	29.4 (±4.6)	5	30.6 (±5.7)
		30.0 [25.5, 33.0]		30.0 [25.3, 33.0]		31.0 [27.0, 36.0]
Prenatal visits	67	2.0 (±0.8)	62	2.0 (±0.8)	5	2.0 (±1.0)
		2 [1, 2]		2 [1, 2]		2 [1, 3]
Prenatal genetic testing						
NIPT	22		22		0	
Normal		12 (55%)		12 (55%)		0 (0%)
Abnormal		10 (45%)		10 (45%)		0 (0%)
Amniocentesis	8		7		1	
Normal		4 (50%)		3 (43%)		1 (100%)
Abnormal		4 (50%)		4 (57%)		0 (0%)
Postnatal genetic testing	57		52		5	
Normal		44 (77%)		39 (75%)		5 (100%)
Abnormal*		13 (23%)		13 (25%)		0 (0%)
GA at birth (weeks)	67	37.8 (±1.8)	62	37.8 (±1.8)	5	38.6 (±1.1)
		38.4 [37.1, 39.0]		38.4 [37.0, 39.0]		39.0 [38.9, 39.3]
Age at surgery (days)	60	12.7 (±14.8)	56	13.6 (±15.2)	5	3.6 (±3.3)
		7 [5, 12]		7 [5, 12.5]		3 [1, 7]

Mean (standard deviation); Median [interquartile range]; AA = African American; US = Ultrasound; GA = Gestational age; CHD = Congenital heart disease; NIPT = Non-invasive prenatal testing

* not including variants of undetermined significance

**Table 2.** Fetal echocardiographic parameters by surgical outcome

	n	Total n = 67	n	Biventricular repair n = 62	n	SVR cardiac death or OHT n = 5

Mitral valve z-score	67	−2.07 (1.01)	62	−1.92 (0.84)	5	−3.98 (1.09)
		−2.18 [−2.55, −1.55]		−2.10 [−2.49, −1.46]		−4.30 [−4.63, −3.13]
Mitral valve anomaly	67		62		5	
No anomaly		56 (83.6%)		55 (88.7%)		1 (20%)
Anomaly*		11 (16.4%)		7 (11.3%)		4 (80%)
Left ventricle width z-score	61	−1.44 (0.99)	56	−1.51 (0.82)	5	−0.67 (2.18)
		−1.46 [−1.98, −0.95]		−1.46 [−1.94, −0.97]		−1.62 [−2.12, 1.46]
Left ventricle length z-score	66	−0.91 (0.72)	61	−0.92 (0.68)	5	−0.83 (1.17)
		−0.88 [−1.37, −0.47]		−0.84 [−1.32, −0.47]		−0.93 [−1.69, −0.79]
Aortic valve z-score	67	−1.67 (0.93)	62	−1.60 (0.90)	5	−2.54 (0.97)
		−1.69 [−2.31, −1.16]		−1.66 [−2.24, −1.06]		−2.55 [−3.06, −1.80]
Ascending aorta z-score	54	−1.89 (1.14)	49	−1.83 (1.07)	5	−2.48 (1.79)
		−1.65 [−2.35, −1.16]		−1.62 [−2.34, −1.15]		−1.93 [−2.68, −1.68]
Isthmus sagittal z-score	26	−2.55 (1.05)	24	−2.56 (1.08)	2	−2.38 (0.47)
		−2.53 [−3.15, −2.09]		−2.53 [−3.18, −2.12]		−2.38 [−2.54, −2.21]
Isthmus 3-vessel z-score	21	−2.76 (1.39)	19	−2.64 (1.41)	2	−3.85 (0.38)
		−2.73 [−3.56, −2.43]		−2.72 [−3.52, −2.40]		−3.85 [−3.98, −3.71]
Isthmus to ductus ratio	20	0.60 (0.07)	18	0.60 (0.08)	2	0.56 (0.06)
		0.60 [0.56, 0.63]		0.60 [0.57, 0.66]		0.56 [0.54, 0.58]
Aortic to pulmonary valve ratio	66	0.62 (0.09)	61	0.62 (0.09)	5	0.54 (0.05)
		0.60 [0.56, 0.67]		0.60 [0.57, 0.68]		0.54 [0.53, 0.58]
Left to right ventricular length ratio	65	0.96 (0.09)	60	0.96 (0.08)	5	0.93 (0.16)
		0.96 [0.90, 1.00]		0.96 [0.91, 1.00]		0.85 [0.85, 0.94]
Mitral to tricuspid valve ratio	67	0.80 (0.51)	62	0.82 (0.53)	5	0.57 (0.09)
		0.72 [0.64, 0.78]		0.74 [0.65, 0.79]		0.58 [0.54, 0.64]
Left superior vena cava	67		62		5	
Absent		54 (80.6%)		50 (80.6%)		4 (80.0%)
Present		13 (19.4%)		12 (19.4%)		1 (20.0%)
Foramen ovale	65		60		5	
Right to left		43 (64.2%)		43 (69.4%)		0 (0%)
Any left to right		22 (32.8%)		17 (27.4%)		5 (100%)

Mean (standard deviation), Median [interquartile range], SVP = single ventricle palliation, OHT = orthotopic heart transplant, n = number of patients with the measurement or finding

* Including morphological anomalies or dysfunction (not including isolated hypoplasia)

**Table 3.** Mitral valve findings for fetuses with CoA who experienced SVP, cardiac death or OHT

	Fetal				Postnatal			
	
	LV & AV Description	GA	MV z-score	MV Description	LV & AV Description	MV z-score	MV Description	Clinical Outcome

1	Mild LV hypoplasia, AS	31.4 35.0	−5.27 n/a	Hypoplasia only	Mild LV hypoplasia, BAV	−2.40	Parachute	BVR; death at 1 year post MV revision
2	Borderline LV hypoplasia, AS	27.0 30.7 35.0	−4.40 n/a −5.00	Parachute, thickened leaflets	Mild LV hypoplasia, normal AV	−1.20	Supramitral ring, long chordae, asymmetric papillary muscles	SVP; death following stage 1 palliation
3	Distended LV, EFE, AS	36.7	−4.47	Moderate insufficiency	Critical AS, distended LV, EFE	−2.00	Arcade, asymmetric papillary muscles, restriction, moderate insufficiency	Death following aortic valvuloplasty
4	Normal LV size, EFE, AS	36.7	−2.75	Moderate to severe insufficiency	Critical AS, mild LV hypoplasia, EFE	−0.41	Severe insufficiency	OHT following aortic valvuloplasty
5	Borderline LV hypoplasia, AS	23.0 28.9 33.9	−3.30 −3.20 −3.00	Thickened, restricted leaflets	Moderate LV hypoplasia, BAV	−3.90	Hypoplasia only	SVP; OHT for failed Fontan physiology

CoA = coarctation of the aorta, SVP = single ventricle palliation, OHT = orthotopic heart transplant, LV = left ventricle, AV = aortic valve, GA = gestational age, MV = mitral valve, AS = aortic stenosis, MS = mitral stenosis, BAV = bicuspid aortic valve, BVR = biventricular repair, EFE = endocardial fibroelastosis

## References

[1] Buyens A, Gyselaers W, Coumans A, (2012) Difficult prenatal diagnosis: fetal coarctation. Facts Views Vis ObGyn. 4(4):230–236. 24753914 PMC3987479

[2] Kaplinski M, Cohen MS (2015) Characterising adequacy or inadequacy of the borderline left ventricle: what tools can we use? Cardiol Young. 25(8):1482–1488. 10.1017/S1047951115002267 26675594

[3] Jantzen DW, Gelehrter SK, Yu S, Donohue JE, Fifer CG (2015) Echocardiographic factors discriminating biventricular versus univentricular approach in the foetus with borderline left ventricle. Cardiol Young. 25(5):941–950. 10.1017/S1047951114001449 25115769

[4] Weber RW, Ayala-Arnez R, Atiyah M, (2013) Foetal echocardiographic assessment of borderline small left ventricles can predict the need for postnatal intervention. Cardiol Young. 23(1):99–107. 10.1017/S1047951112000467 22475329

[5] Haberer K, Fruitman D, Power A, Hornberger LK, Eckersley L (2021) Fetal echocardiographic predictors of biventricular circulation in hypoplastic left heart complex. Ultrasound Obstet Gynecol. 58(3):405–410. 10.1002/uog.23558 33270293

[6] Edwards LA, Arunamata A, Maskatia SA, (2019) Fetal Echocardiographic Parameters and Surgical Outcomes in Congenital Left-Sided Cardiac Lesions. Pediatr Cardiol. 40(6):1304–1313. 10.1007/s00246-019-02155-7 31338561

[7] Colquitt JL, Loar RW, Bolin EH, Ezon DS, Heinle JS, Morris SA (2022) Left heart hypoplasia in the fetus: Echocardiographic predictors of outcome. PrenatDiagn. 42(4):447–460. 10.1002/pd.6101 35040508

[8] Viaux-Savelon S, Dommergues M, Rosenblum O, (2012) Prenatal Ultrasound Screening: False Positive Soft Markers May Alter Maternal Representations and Mother-Infant Interaction. PLOS ONE. 7(1):e30935. 10.1371/journal.pone.0030935 22292077 PMC3264650

[9] Parameter(z). Accessed June 13, 2023. http://parameterz.blogspot.com/

[10] R Workflow - 13 Manipulation of Longitudinal Data. Accessed June 7, 2023. https://hbiostat.org/rflow/long.html#sec-long-interp

[11] Toole BJ, Schlosser B, McCracken CE, Stauffer N, Border WL, Sachdeva R (2016) Importance of Relationship between Ductus and Isthmus in Fetal Diagnosis of Coarctation of Aorta. Echocardiography. 33(5):771–777. 10.1111/echo.13140 26667892

[12] Kailin JA, Santos AB, Yilmaz Furtun B, Sexson Tejtel SK, Lantin-Hermoso R (2017) Isolated coarctation of the aorta in the fetus: A diagnostic challenge. Echocardiography. 34(12):1768–1775. 10.1111/echo.13578 29287141

[13] Quartermain MD, Cohen MS, Dominguez TE, Tian Z, Donaghue DD, Rychik J (2009) Left Ventricle to Right Ventricle Size Discrepancy in the Fetus: The Presence of Critical Congenital Heart Disease Can Be Reliably Predicted. J Am Soc Echocardiogr. 22(11):1296–1301. 10.1016/j.echo.2009.08.008 19815386

[14] Jowett V, Aparicio P Santhakumaran S, Seale A, Jicinska H, Gardiner HM (2012) Sonographic predictors of surgery in fetal coarctation of the aorta: Sonographic predictors of fetal CoA. Ultrasound Obstet Gynecol. 40(1):47–54. 10.1002/uog.11161 22461316

[15] Fricke K, Liuba P Weismann CG (2021) Fetal Echocardiographic Dimension Indices: Important Predictors of Postnatal Coarctation. Pediatr Cardiol. 42(3):517–525. 10.1007/s00246-020-02509-6 33355680 PMC7990842

[16] Conte S, Lacour-Gayet F, Serraf A, (1995) Surgical management of neonatal coarctation. J Thorac Cardiovasc Surg. 109(4):663–675. 10.1016/S0022-5223(95)70347-0 7715213

[17] Feinstein JA, Benson DW, Dubin AM, Hypoplastic Left Heart Syndrome (2012) J Am Coll Cardiol. 59(1_Supplement):S1–S42. 10.1016/j.jacc.2011.09.022 22192720 PMC6110391

[18] Sharland GK, Chita SK, Fagg NL, (1991) Left ventricular dysfunction in the fetus: relation to aortic valve anomalies and endocardial fibroelastosis. Heart. 66(6):419–424. 10.1136/hrt.66.6.419 PMC1024814 1837727

[19] McCaffrey FM, Sherman FS (1997) Prenatal Diagnosis of Severe Aortic Stenosis. Pediatr Cardiol. 18(4):276–281. 10.1007/s002469900174 9175524

[20] Axt-Fliedner R, Kreiselmaier P Schwarze A, Krapp M, Gembruch U (2006) Development of hypoplastic left heart syndrome after diagnosis of aortic stenosis in the first trimester by early echocardiography. Ultrasound Obstet Gynecol. 28(1):106–109. 10.1002/uog.2824 16795135

[21] Allan LD, Sharland G, Tynan MJ (1989) The natural history of the hypoplastic left heart syndrome. Int J Cardiol. 25(3):341–343. 10.1016/0167-5273(89)90226-X 2613383

[22] Walsh MJ, Verghese GR, Ferguson ME, (2017) Counseling Practices for Fetal Hypoplastic Left Heart Syndrome. Pediatr Cardiol. 38(5):946–958. 10.1007/s00246-017-1601-1 28345115

[23] Luo JL, Zhao BW, Pan M, (2020) Z-scores of early diastolic blood flow widths of mitral and tricuspid valves in normal fetuses and fetuses with dilated coronary sinus. J Matern Fetal Neonatal Med. 33(9):1579–1586. 10.1080/14767058.2018.1523894 30238805

[24] Gu X, Zhu H, Zhang Y, (2019) Quantile Score: A New Reference System for Quantitative Fetal Echocardiography Based on a Large Multicenter Study. J Am Soc Echocardiogr. 32(2):296–302.e5. 10.1016/j.echo.2018.09.012 30591282

